# Adverse Health Effects Related to Shift Work Patterns and Work Schedule Tolerance in Emergency Medical Services Personnel: A Scoping Review

**DOI:** 10.7759/cureus.23730

**Published:** 2022-04-01

**Authors:** Jennifer Barth, Jennifer A Greene, Judah Goldstein, Aaron Sibley

**Affiliations:** 1 Division of Paramedicine, Department of Biology, University of Prince Edward Island, Charlottetown, CAN; 2 Division of Emergency Medical Services, Department of Emergency Medicine, Dalhousie University, Halifax, CAN

**Keywords:** stress, sleep, fatigue, health, shift work tolerance, shift work, work schedule, paramedicine, emergency medical services

## Abstract

Paramedicine as a profession is continually evolving in clinical practice, responsibilities, and workload. Changes over time in both population demographics and distribution have altered the demand for, and availability of, prehospital emergency medical services (EMS). These factors may also affect scheduling policies in many EMS organizations. However, there is little evidence that suggests optimal shift scheduling patterns to reduce adverse health events such as increased stress or fatigue in prehospital emergency health care providers. Our objective was to describe associations between variations in shift scheduling patterns and EMS provider health outcomes, such as fatigue, stress, sleep quality, and general mental and physiological health. We also sought to identify knowledge gaps. We performed searches of PubMed, CINAHL, Embase, and Cochrane databases for primary studies, systematic reviews, and meta-analyses published between January 2000 and December 2020. Studies reporting measurable health care outcomes in prehospital personnel with defined shift schedule patterns in land-based ambulance systems were included. Our search strategy yielded 188 studies, of which 11 met eligibility criteria (eight cross-sectional surveys, one single case report, one retrospective cohort study, one prospective cohort study, and one systematic review), with one additional study found through reference list screening, leaving 12 studies for review. All publications contained a description of shift schedule characteristics and shared similar outcomes of interest, although there was variation in comparators and assessment of outcomes. Most studies showed high rates of fatigue, stress, mental health concerns, and negative general health outcomes in paramedic shift worker populations. The case study reported improved fatigue, alertness, and sleep quality levels following a switch from a 24-hour shift pattern to an eight-hour shift. We did not complete an in-depth risk of bias assessment for any of the studies. Melnyk evidence ratings varied from IV to VI, indicating a low quality of evidence evaluating the impacts of shift schedule patterns in paramedics, with the retrospective cohort study design, ranked as IV, systematic review as a V, and prospective cohort study, case report and surveys ranked as VI. The low quality and quantity of evidence indicate the need for further research to definitively assess relationships between specific schedule patterns and health outcomes.

## Introduction and background

Fatigue, stress, and poor health have been associated with shift work, both in health care workers and the general population [1-3]. Shift workers in both the general public and health care workers such as nurses and emergency medical services (EMS) personnel have been shown to have more negative health outcomes, such as increased fatigue and sleepiness, and decreased mental and physical health [4,5]. However, the prehospital health care environment has unique areas of responsibility above and beyond clinical patient care, including safe emergency vehicle operation and compliance with public safety standards. Due to the importance of provision of high-quality emergency medical care in the prehospital environment, it is critical to understand the effects of shift work and shift schedules on EMS personnel health.

Shift work is essential in areas where services are in demand around the clock, such as the health care sector [6]. In order to provide an effective level of medical service to the public, EMS systems utilize a variety of shift schedule patterns. Specific shift scheduling practices may include consecutive or rotating patterns of day or night shifts of varying shift durations, often between eight and 12 hours or up to 24 or 48 hours. 

Though there is existing research related to fatigue risk management, sleep quality, overall health, and certain aspects of shift work such as shift duration in the prehospital workplace environment, there is a limited amount addressing associations between specific shift schedule patterns with these outcomes [7-9]. Rotating shift schedules have been linked to decreased job performance in the health care sector [10], while recent studies examining shift schedule patterns such as a “two day, two night, four off” identified increasing sleepiness and an increased risk of fatigue-related errors in doctors as consequences of shift work [11], as well as a higher likelihood of being involved in motor vehicle accidents in other emergency service personnel such as police officers [12].

Variable quality of sleep and its corollary health changes have been associated with shift work and its stressors [13], with EMS personnel susceptible to increasing stress levels, mental health disorders, decreased neurocognitive performance, fatigue, and sleep disruption that may further exacerbate poor sleep quality and overall health [6]. Shift work has been associated with impaired sleep and decreased quality and duration of sleep [13] and significant sleep, stress, and fatigue level changes within the rotation of day and night shifts in EMS [14]. Further to this, studies assessing the impact of rotational day and night shift work in EMS have found direct links between levels of fatigue and safety outcomes, such as injuries and safety-compromising behaviors [15].

The objective of this study was to map the evidence and highlight knowledge gaps related to adverse health effects associated with shift scheduling patterns in EMS. This review investigated whether variations in shift scheduling patterns affect self-reported stress, fatigue, sleepiness, and sleep cycle changes in emergency medical service professionals. We aimed to highlight associations between shift patterns and health outcomes to inform future areas of research to determine the most appropriate shift patterns that balance system and personnel needs.

This study was previously presented at Dalhousie University’s 13th Annual EMS Research Day on October 14, 2021.

## Review

Methods

We conducted this scoping review in accordance with the Joanna Briggs Institute methodology for scoping reviews [16] and registered the a priori objectives, criteria, and methods with Open Science Framework (OSF) [17]. To determine the need for a scoping review on this topic, we performed a preliminary search of PubMed to discover individual articles on the area of interest, followed by a search of PROSPERO to identify any systematic, rapid, or umbrella reviews on the topic. We were unable to find any scoping or systematic reviews on the specific area of interest. 

*Inclusion and Exclusion Criteria* 

We included studies involving EMS personnel (e.g., paramedics, emergency medical technicians, etc.) in land-based ambulance systems employed in shift work of varying scheduling patterns or shift rotations. Included studies must have described specific shift schedule patterns of any variation, as well as health-related outcomes, such as self-reported levels of stress, fatigue, sleepiness, sleep cycle, or physiological health changes. We included studies using both primary (randomized control trials, cohort studies, cross-sectional studies, and case-control) and secondary (systematic reviews and meta-analyses) research designs. We excluded gray literature, abstracts, theses, conference proceedings, and opinion papers.

Search Strategy

On December 7, 2020, we conducted a search of four databases: PubMed, CINAHL, Embase, and Cochrane. For this study, the search incorporated concepts and terms related to three overlapping areas: EMS or prehospital personnel such as paramedics; shift work, shift tolerance, or shift work schedule; and fatigue and other fatigue-related outcomes such as sleep, sleep changes, stress, and health changes. We included only English articles published between January 2000 and December 7, 2020.

Study Selection

We exported the search results into Covidence, with duplicates noted and removed [18]. We assessed studies for inclusion via title and abstract screening by two independent reviewers (JB and JAG). If a decision could not be reached from title and abstract screening, we obtained the full-text document to determine relevance. Four reviewers (JB, JAG, JG, and AS) evaluated full-text articles for eligibility in pairs of two, with conflicts adjudicated by the senior reviewer (AS). Finally, we searched the reference lists of each included study for relevant missed publications that we evaluated for study inclusion.

Data Extraction

We developed a data extraction table to identify key data points from each selected study for summary and analysis. Data extracted included primary study author, date, location, study type and design, duration, personnel type, gender, shift schedule details, comparator details if applicable, outcomes measured in each study, survey tools used, survey response rate, and findings of interest (Table 1). All reviewers participated in data extraction (JB, JAG, JG, and AS) in rotating pairs to ensure complete and accurate collection.

**Table 1:** Data extraction form EMT: emergency medical technician; PCP: primary care paramedic; ACP: advanced care paramedic

Author/year of publication	Location (city, region, country)	Design	Melnyk scale score	Size (number of participants)	Gender of respondents (M%:F%)	Date/duration	Survey/study tools	Survey response rate
Author/year of publication	Population/participants (EMT, PCP, ACP, first responder)	Shift schedule intervention pattern in population of interest		Comparator description (if applicable)		Outcomes of interest		Findings

Data Synthesis

Findings are presented in accordance with the Preferred Reporting Items for Systematic reviews and Meta-Analyses extension for Scoping Reviews (PRISMA-ScR) checklist [19]. Findings related to the objectives and research question are presented individually or as part of an identified subsection of grouped studies, involving similar outcomes of specific schedules or scheduling practices. Health-related outcomes from studies were grouped by shift schedule pattern, based on the type of shift schedule pattern described in the study. Groupings were determined by the majority type of shift schedule pattern in the study, whether a series of rotating day and night shifts, or a more even mix of rotating day and night shifts and fixed shift patterns of either day or night shifts. A cut-off value of 75% was applied to determine the assigned grouping of each study, based on the percentage of study participants working either a rotating or more even mix of shift schedule patterns. A case study involving a shift schedule switch was grouped separately for discussion.

Methodological Quality Appraisal

We used the Melnyk and Fineout-Overholt classification scale to rank level of evidence of included studies. Studies are ranked based on design as well as the source of data in inclusive levels of I-VII. Systematic reviews or meta-analysis of randomized controlled trials rank as level I, followed by single randomized controlled trials (level II), experimental studies and/or non-randomized controlled trials (level III), cohort or case-control studies (level IV), systematic reviews or meta-analysis of qualitative studies (level V), single qualitative or descriptive studies or evidence implementation and quality improvement projects (level VI), and expert opinion (level VII) [20]. 

Results

Search Strategy Results

**Figure 1:**
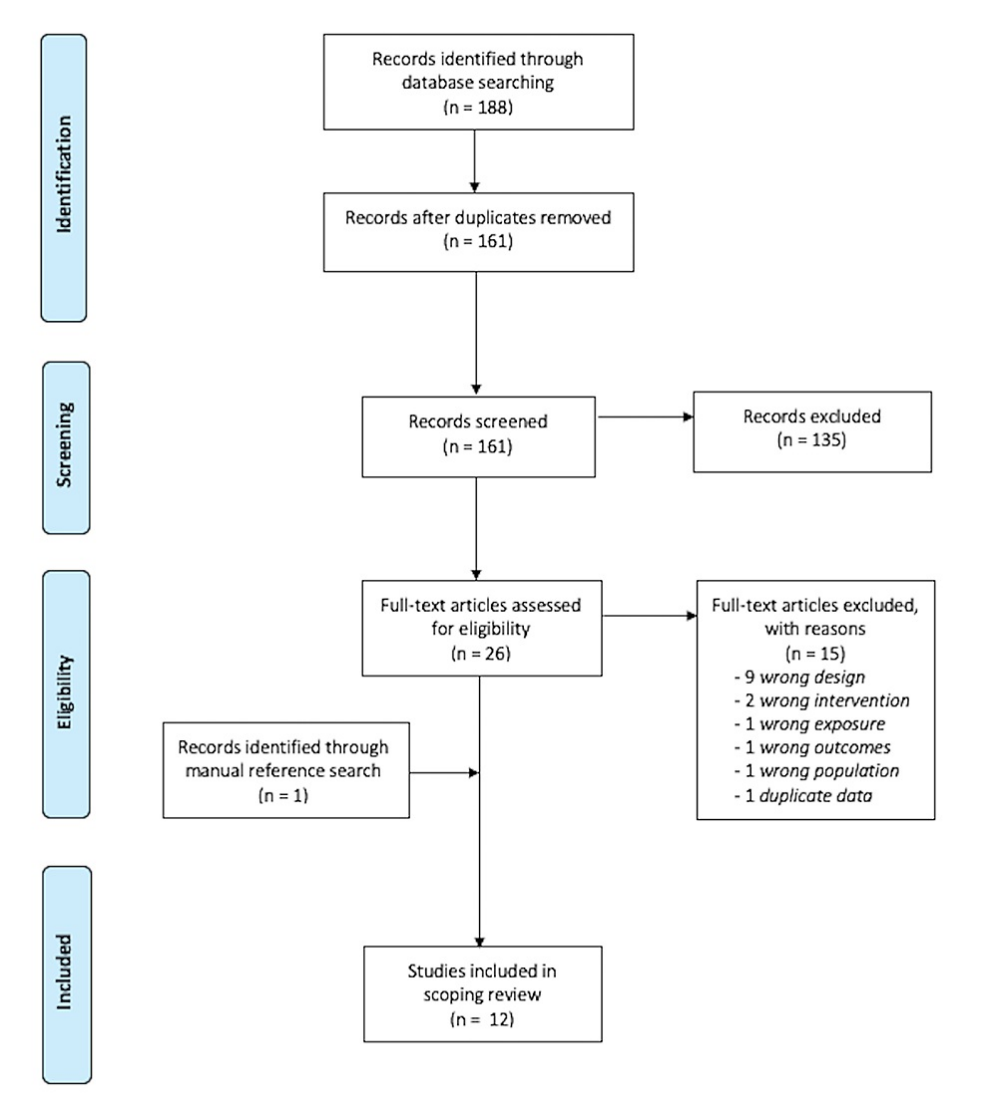
PRISMA-ScR flow diagram PRISMA-ScR: Preferred Reporting Items for Systematic reviews and Meta-Analyses extension for Scoping Reviews

The search strategy yielded 188 results, including 27 duplicates, leaving a total of 161 studies for initial screening (Figure 1). We excluded 135 studies following title and abstract screening, leaving 26 for full-text screening. We excluded 15 studies based on full-text review, most commonly due to wrong study design (n = 9), leaving 11 studies. Following reference list screening, one additional study was added, leaving 12 studies for data extraction and analysis (Figure 1).

Characteristics of Included Studies

The majority (n = 11) of included studies were observational studies, in addition to one systematic review (Table 2). Of the observational studies, eight were cross-sectional surveys [4,5,14,15,21-24], with one single case report [25], one retrospective cohort study [26], and one prospective cohort study [27]. Surveys yielded the largest sample sizes: four above 100 participants [5,21-23], two above 300 [4,24], and one above 700 [15], though one cross-sectional survey included only 12 participants [14]. The proportion of study participants identified as female sex ranged between 26.5% and 58%, while one study included only male participants.

**Table 2:** Characteristics of included studies OSHA: Occupational Safety and Health Administration; n.d.: no date; EMS: emergency medical services

Author/year	Location (city, region, country)	Design	Date/duration	Sample size (n)	Sex (M%:F%)
Courtney et al., 2010 [4]	Melbourne, Victoria, Australia	Cross-sectional survey	July 2006 to November 2006	342	(71%:29%)
Courtney et al., 2013 [21]	Bundoora, Victoria, Australia	Cross-sectional survey	Single time point (n.d.)	150	(73.5%:26.5%)
Donnelly et al., 2019 [15]	Ontario, Canada	Cross-sectional survey	2015; 3 months (n.d.)	717	(66%:34%)
Khan et al., 2020 [22]	Makkah District, Western region, Saudi Arabia	Cross-sectional survey	AU sample: December 2017 to December 2018; SA sample: November 2018 to January 2019	121	(100%:0%)
Khan et al., 2020 [23]	State of Victoria, Australia	Cross-sectional survey	December 2017 to December 2018	134	(45.8%:54.2%)
Khan et al., 2021 [14]	State of Victoria, Australia	Cross-sectional survey	8 days per participant in study; (n.d.)	12	(42%:58%)
Patterson et al., 2010 [5]	Pennsylvania, USA	Cross-sectional survey	2 days; 2008 (n.d.)	119	(46%:54%)
Patterson et al., 2015 [24]	Pennsylvania, USA	Cross-sectional survey	February 2014 to March 2014	423	(61%:39%)
Patterson 2016 [25]	Pennsylvania, USA	Single case-control study	January 2014 to June 2014	1	(100%:0%)
Patterson et al., 2018 [7]	Pittsburgh, Pennsylvania, USA	Systematic review	2016	100	N/A
Weaver et al., 2015 [26]	Boston, Massachusetts, USA	Retrospective cohort study	2015	14 EMS systems (966,082 shifts; 960 (OHSA reports)	N/A
Wong et al., 2012 [27]	Vancouver, British Columbia, Canada	Prospective cohort study	November 2008 to January 2009	21	(67%:33%)

Of 12 included publications, 10 were graded as level VI due to their design as either single studies or qualitative studies, as well as variation in their subjective assessment tools. We ranked the systematic review as level V, as it assessed descriptive and qualitative studies. We assigned our highest ranked prospective cohort study as level IV, due to its structured design and sampling assessment methodology.

All studies included land-based EMS personnel as the population of interest; comparators included EMS personnel [5,7,21,22,24,27], nurses [21], industrial shift workers [21], and general population sampling [5,23]. The systematic review captured three of the individual studies included in this review [24-26]. All articles offered a shift schedule description; six studies included a minimum of 75% of participants working on a rotating shift pattern including day and night shifts (Table 3), five studies including a mix of rotating day and night shifts worked by less than 75% of participants together with fixed shift patterns of varying length of either day or night shifts (Table 4), and one case report involving a direct comparison between two different shift rotations in which shift length influenced shift schedule pattern (Table 5).

**Table 3:** Health-related outcomes in EMS personnel working rotating shift patterns GH: general health; PTSD: post-traumatic stress disorder; TST: total sleep time; HRV: heart rate variability; EMS: emergency medical services

Author/year	Shift schedule (% of sample working shift pattern)	Outcomes	Findings
Courtney et al., 2010 [4]	2 day (10h)/2 night (14h) roster: 80%; “nightshift rotation”: 20%	Sleep quality; fatigue; mental health; physical activity	Significantly higher fatigue, depression, anxiety, stress; significantly higher rate of poor sleep quality; 14% less physical activity
Donnelly et al., 2019 [15]	Rotating shifts between day/nights: (75.7%)	Levels of fatigue; safety outcomes: injury, safety compromising behaviors	55% reported fatigue; 33.5% reported injury; 96.2% reported safety compromising behavior; rotating shifts approached significant levels of injury prediction (p = 0.077)
Khan et al., 2020 [22]	2 day shifts (12h), 2 night shifts (12h), 3 days off (95%); fixed schedule of 4 day shifts or 4 night shifts (5%)	General health; stress; depression; anxiety; sleep; sleepiness; fatigue	Significantly higher depression, PTSD, insomnia and poorer GH in SA sample compared to AU sample
Khan et al., 2020 [23]	2 day shifts (10h), 2 night shifts (14h), 4 days off (85.4%); fixed schedule of 4 day shifts (10h) or 4 night shifts (14h), 4 days off (7.6%); 8 consecutive day shifts (10h), overnight on call, 6 days off (7.0%)	Stress; depression; anxiety; sleep; sleepiness; fatigue	Significantly higher rates of depression, poor sleep quality, anxiety, PTSD, insomnia, fatigue and narcolepsy compared to general population samples
Khan et al., 2021 [14]	2 day shifts (10h), 2 night shifts (14h), 4 days off	Total sleep time; sleepiness; stress; mood; fatigue; physical activity	TST, sleepiness, stress and physical activity levels varied significantly across rotation; fatigue levels varied depending on assessment time
Wong et al., 2012 [27]	14 rotating shift work schedules (5 dispatch, 9 ambulance paramedics)	Salivary α-amylase; salivary cortisol; heart rate variability; endothelial dysfunction	Compared to fixed schedule (day) shift workers, rotating shift workers reported: ・Higher job strain; flatter α-amylase and cortisol diurnal slopes; reduced daily α-amylase production; elevated daily cortisol production; reduced HRV; reduced endothelial function

**Table 4:** Health-related outcomes in EMS personnel working rotating and fixed shift patterns combined OSHA: Occupational Safety and Health Administration; EMS: emergency medical services; SR: systematic review

Author/year	Shift schedule (% of sample working shift pattern)	Outcomes	Findings
Courtney et al., 2013 [21]	2 day (10h)/2 night (14h) roster: 50%); “nightshift component”: 50%	Sleep quality; fatigue; mental health; physical activity	No significant difference for depression, anxiety, stress; 2.5% less physical activity
Patterson et al., 2010 [5]	<8h shifts: 21.2% (24); 8h shifts: 35.4% (40); 12h shifts: 28.3% (32); 24h shifts: 15.0% (17)	Sleep; fatigue; health	Overall severe fatigue levels (45%), related to poor sleep quality
Patterson et al., 2015 [24]	<12h shift (33%); 12h shift (29%); >12h shift/“other” (38%)	Health; sleep; fatigue; sleepiness; affect	Inter-shift recovery higher for >12h shift schedules (33%), lowest for 12h shifts (29%); moderate to high levels of inter-shift recovery was highest for >12h shifts (61.6%), lower in <12h shifts/“other” shifts (47.7%), lowest in 12h shifts (40.2%)
Patterson et al., 2018 [7]	Shift duration (8h, 12h, 24h)	Critical: patient safety; personnel safety important: acute fatigue; sleep; sleep quality; long-term health; burnout/stress	Conclusions from SR focused primarily on shift duration and fatigue in EMS personnel; noted quality of existing evidence low or very low; conclusions from three applicable studies from SR are discussed elsewhere: Patterson et al., 2015 [24]; Patterson et al., 2016 [25]; Weaver et al., 2015 [26]
Weaver et al., 2015 [26]	Shift length variations of ≤8h >12h and ≤16h and >16h and ≤24h	Occupational Safety and Health Administration (OSHA) reported injury or illness	Shifts >12h and ≤16h increased risk injury by 27%; shifts >16h and ≤24h increased risk injury by 60%; shifts ≤8h decreased risk injury by 30%

**Table 5:** Health-related outcomes from a single case study assessing shift pattern change EMS: emergency medical services

Author/year	Shift schedule	Outcomes	Findings
Patterson et al., 2016 [25]	EMS system utilizing 8h shift rotations vs. 24h shift rotation	Sleep quality; fatigue; health; sleepiness; concentration (alertness)	Sleep quality score improved from baseline to 90 days following switch from 24h rotation to 8h rotation; sleepiness level dropped from “excessive sleepiness” to “situational sleepiness”; chronic and acute fatigue levels improved; no difference between sleep quantity prior to start of shift between 24h and 8h shifts; possible increase in intent to engage in alertness behaviors while at work; improved or strengthened perception of working while fatigued; improved self-efficacy/self-confidence in engaging in behaviors that may improve alertness; decrease over time in attitude/perspective towards maintaining alertness while at work

**Figure 2:**
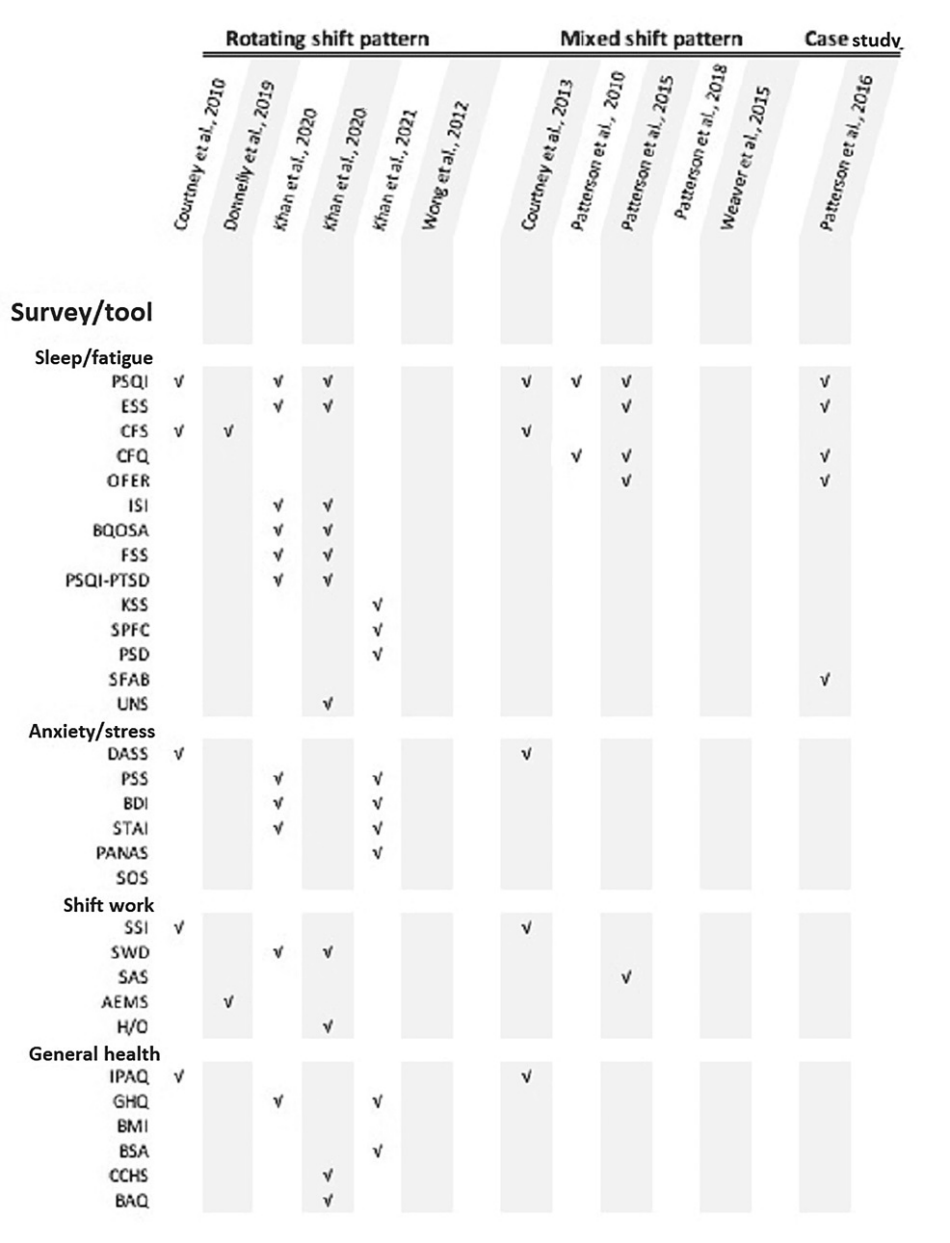
Survey/tool used in studies grouped by shift pattern type Figure created by the authors of this study. PSQI: Pittsburgh Sleep Quality Index; ESS: Epworth Sleepiness Scale; CFS: Chronic Fatigue Scale; CFQ: Chalder Fatigue Questionnaire; OFER: Occupational Fatigue Exhaustion Recovery scale; ISI: Insomnia Severity Index; BQOSA: Berlin questionnaire-obstructive sleep apnea; FSS: Fatigue Severity Scale; PSQI-PTSD: Pittsburgh Sleep Quality Index-posttraumatic stress disorder; KSS: Karolinska Sleepiness Scale; SPFC: Samn-Parelli Fatigue Checklist; PSD: Pittsburgh Sleep Diary; SFAB: Sleep Fatigue Alertness Behavior Survey; UNS: Ullanlinna Narcolepsy Scale; DASS: Depression Anxiety Stress Scales; PSS: Perceived Stress Scale; BDI: Beck Depression Inventory; STAI: State-Trait Anxiety Inventory; PANAS: Positive Affect and Negative Affect Scale; SOS: Sources of Occupational Stress Survey; SSI: Standard Shiftwork Index; SWD: Shiftwork Disorder Screening Questionnaire; SAS: Schedule Attitudes Survey; AEMS: American Emergency Medical Services Survey; H/O: Horne and Ostberg questionnaire for chronotype; IPAQ: International Physical Activity Questionnaire; GHQ: General Health Questionnaire; BMI: body mass index; BSA: BodyMedia SenseWear Armband; CCHS: Canadian Community Health Survey; BAQ: Bruxism Assessment Questionnaire

Outcomes in each study varied; all but one study included fatigue, stress, and health changes predominantly assessed by subjective, qualitative assessment scales. Validated scales used to measure outcomes in these studies were chosen by the study authors, resulting in a wide variety of survey tools assessing overlapping outcomes. Of all survey tools identified, the three most common were the Pittsburgh Sleep Quality Index (PSQI), used in seven of the 12 publications [4,5,21-25], followed by the Epworth Sleepiness Scale (ESS), used in four [22-25], and the Chronic Fatigue Scale (CFS) [4,15,21] and the Chalder Fatigue Questionnaire (CFQ) [5,24,25], each used in three (Figure 2). One study, focusing on physiological health factors quantifiable by salivary sampling and heart rate variability, used Polar RS 800 Heart Rate wireless monitors (Kempele, Finland: Polar Electro Oy) and the EndoPAT2000 device (Caesarea, Israel: Itamar Medical Ltd.) for non-invasive assessment of peripheral arterial tone [27].

Health-Related Outcomes in EMS Personnel Working Rotating Shift Patterns

Studies comprised of a high percentage of participants (≥75%) working a rotating shift pattern showed high rates of fatigue and poor sleep quality compared to nursing and industrial shift workers [5], as well as high rates of insomnia, depression, post-traumatic stress disorder (PTSD), and anxiety (Table 2) [22,23]. A Canadian study focusing on the relationship between fatigue and safety-compromising behavior in EMS personnel found that 55% of participants reported fatigue while at work and 96.2% reported safety-compromising behavior, although the relationship was not statistically significant [16]. Another Canadian study examining hormone changes and physiological responses to shift work in paramedics found that in comparison to fixed day or night shifts, rotating shifts patterns were associated with higher job strain, elevated cortisol production, reduced alpha-amylase production, reduced heart-rate variability (HRV), and reduced endothelial function [27].

Health-Related Outcomes in EMS Personnel Working Rotating and Fixed Shift Patterns Combined

Studies with EMS personnel working a more even mix of shift schedule patterns reported overall severe fatigue levels related to poor sleep quality [6], but no significant differences in depression, anxiety, and stress when compared to the general population and nursing or industrial shift workers (Table 3) [21]. An American study comparing rest recovery rates between shift patterns of different shift durations found that moderate to high levels of inter-shift recovery was highest for shifts over 12 hours in length (61.6%), lower for shifts under 12 hours in length (47.7%), and lowest shifts for 12 hours (40.2%) [24]. Injury risk associated with shift pattern based on shift length increased in shifts over 16 hours and under 24 hours in length by 60%, increased in shifts over 12 hours and under 16 hours in length by 27%, but decreased by 30% in shifts under to or equal to 8 hours in length [26].

Health-Related Outcomes From a Single Case Study Assessing Shift Pattern Change

An American study comparing sleep, fatigue, and alertness levels in a single participant following a switch from a shift schedule pattern utilizing a 24-hour shift to a pattern based on eight-hour shifts found chronic fatigue, acute fatigue, and sleep quality scores improved from baseline rates when assessed at 90 days, while reported sleepiness levels dropped from "excessive sleepiness" to "situational sleepiness" (Table 4) [25]. "Excessive sleepiness" and "situational sleepiness" assessments were determined by the participant’s pre-pattern switch baseline score of 16 and post-pattern switch score of 12 on the Epworth Sleepiness Scale (ESS) [25]. There was no difference noted in sleep quantity prior to start of shifts when the shift schedule pattern based on a 24-hour shift was compared to the pattern based on eight-hour shifts. The participant reported an "improved or strengthened perception of working while fatigued," as well as "improved self-efficacy in engaging in behaviors that may improve alertness" and a possible increase in intent to engage in alertness behavior while at work [25]. However, there was a reported decrease over time in attitude or perspective towards maintaining alertness while at work [25].

Discussion

Summary of Main Results

We found 12 articles reporting on fatigue, stress, sleep quality, and mental and physiological health-related outcomes in EMS personnel working defined shift schedule patterns. Overall, the quality of the evidence assessed using the Melnyk scale was low, with 10 of the 12 studies ranked as level VI. Approximately half of all studies included ≥75% of participants working in rotating shift schedule patterns, while five studies included mixed levels of several different types of shift schedule patterns. All but one of the studies were observational in nature, with only a single case report assessing a controlled shift pattern change from a 24-hour shift to an eight-hour shift rotation that allowed for pre- and post-interventional analysis of outcomes [25].

Health-Related Outcomes in EMS Personnel Working Rotating Shift Patterns

Four studies reported high fatigue, depression, anxiety, and stress in EMS personnel [4,15,22,23] compared to shiftwork studies of nurses and industrial personnel [4] and general population samples [22,23]. Further, three studies observed poor sleep quality, sleepiness, and poor general health in EMS personnel in rotating schedules compared to general population samples [14,22,23]. This is comparable to research evaluating in-hospital nursing populations, reporting increasing levels of sleepiness combined with decreasing levels of inter-shift recovery as the shift schedule progresses [28]. Though there are many confounding variables in each study, including variances in participants, results suggest an association between rotating shift schedule patterns and overall levels of fatigue, sleep, and sleepiness level changes in the overall EMS population, especially in regards to the sequence of consecutive day and night shifts.

Wong et al. found that rotating shift work in paramedics contributed to higher levels of job strain as compared to day shift-only paramedics [27]. Noted increases in the stress hormone cortisol, reduced HRV, and reduced endothelial function indicated a reduction in overall physiological health and immune responses [27]. Shift-working populations that perform non-medical tasks similar to those in EMS, such as truck drivers, also exhibit higher levels of cortisol [27]. Interestingly, unlike in drivers primarily working day shifts, who display higher cortisol levels only during work shifts, cortisol levels remained high even during rest days in drivers who work irregular or rotating shift patterns that included nights [29]. One possible conclusion is that in shift schedule patterns which include both day and night shifts, which are common shift schedules in EMS systems as well, the regularly scheduled rest days are not sufficient to allow hormone levels to restabilize [29].

Health-Related Outcomes in EMS Personnel Working Rotating and Fixed Shift Patterns Combined

Of the five grouped studies reflecting a more even mix of shift patterns, a study by Patterson et al. in 2010 relates overall generalized severe fatigue levels to poor sleep quality in 45% of study participants [5], while a separate study by Courtney et al. in 2013 showed no significant difference in depression, anxiety or stress changes across its prehospital provider population [21]. When investigating the potential for increased risk of injury in relation to shift length, Weaver et al. showed an increased risk of injury can be associated with specific shift lengths: shifts over 12 hours and under 16 hours in length show an increased risk of injury of 27%, while shifts under eight hours in length show a decreased risk of 30% [26].

Inter-shift recovery is an important variable to consider in shift schedule patterns, as fatigue levels can also be evaluated by inter-shift recovery rates. As found by Patterson et al. in 2015, inter-shift recovery rates were highest for shift schedules utilizing shifts over 12 hours in length, lower for those with shifts under 12 hours in length, and lowest for those schedules specifically with 12-hour shifts [24]. Low recovery rates associated with 12-hour shifts requiring sudden changes in the sleep-wake alertness cycle occurred as a result of rotating shift schedules utilizing 12-hour shifts in day/night patterns [24].

In in-hospital nursing populations, 12-hour shifts were also more commonly associated with less total sleep time and lower sleep quality [30]. In EMS personnel, shifts under 12 hours in length tended to involve more gradual transitions between day and night shifts, which had less of an impact on sleep cycle changes and sleep quality [31]. Shift schedule patterns utilizing 12-hour shifts, common in rotating day and night schedules, showed the lowest rate of inter-shift recovery, possibly as a result of the relatively fast changes in sleep cycle and the transition between day and night shifts [28]. This pattern of decreased recovery is also seen in nurses, with increased sleep debt as a result of consecutive 12-hour shifts [28].

Health-Related Outcomes From a Single Case Study Assessing Shift Pattern Change

Patterson et al. showed that sleep quality scores improved from baseline to 90 days following the rotation switch, and sleepiness level dropped from what was described as "excessive sleepiness" to "situational sleepiness" [25]. "Excessive sleepiness" and "situational sleepiness" assessments were determined by the participant’s pre-pattern switch baseline score of 16 and post-pattern switch score of 12 on the Epworth Sleepiness Scale (ESS) [25]. Though there was no difference noted to actual sleep quantity prior to the start of a shift when comparing the two different shifts, the changes related to fatigue levels and overall sleep quality could be associated with the alteration of sleep pattern in between shifts, as changes in shift patterns related to shift length have been attributed to sleep cycle changes [27]. In previous research into shift length and type of rotation, evidence has shown that gradual rotation of shifts across a 24-hour period has a lower detrimental impact on sleep length in rotating shift schedules, while the nights in a rotating shift schedule are associated with a greater negative impact on sleep length than nights in a permanent night shift pattern [32].

Summary of Implications

Research into sleep and wake cycles in safety-critical industries such as marine and land long-haul transport services show that imbalanced ratios of rest:work in favor of rest time are most beneficial for improving overall sleep and sleepiness levels in workers [33]. As assessed by Weaver et al. and Patterson et al., shift length is a component of EMS shift work that affects outcomes related to shift schedule patterns [5,7,24-26]. When directly comparing shift patterns of varying shift lengths, differences in outcomes such as fatigue levels, sleep quality, and alertness behaviors between longer shifts and shorter shifts favor shorter shift length [25].

However, the lack of research into specific shift schedule patterns in EMS does not allow any definitive conclusions to be made concerning the most appropriate shift pattern of either rotating or consecutive day or night shifts in EMS systems. We identified knowledge gaps related to the effects of either fixed or rotating day and night shifts, contributing to a lack of specific understanding of how shift schedule patterns affect the health and wellness of EMS personnel. Initial implications of this review in current practice related to the impact of shift work on EMS personnel is to prioritize consistent fatigue monitoring in employees, with consideration given to shift schedule pattern and shift length in addition to the overall amount of hours worked.

Implications for research related to shift work in EMS include conducting studies assessing shift schedule patterns. Shifts of less than 12 hours in length are shown to be less detrimental to sleepiness, fatigue levels, and general health [14,15,27,32], while also allowing for a work:rest ratio biased in favor of rest [33]. However, there are few studies that directly assess different shift schedule patterns of either consistent or rotating day or night shifts in EMS. The impact of employee health and wellness on EMS system costs related to sick time usage, productivity levels, and employee retention is an important consideration. EMS systems as a whole could benefit from studies specifically addressing the association between shift scheduling patterns, individual employee health outcomes, and EMS system needs.

Limitations

Important limitations should be considered when interpreting the findings of this review. Included studies were of low quality and were not specifically designed to address the research question regarding shift schedule pattern comparisons. There were significant disparities in location of study, sample size, and specific characteristics of participants, as well as a lack of consistent objective measures related to review measures. Variance in the location of each study can create the opportunity for confounding variables such as overall workload, intensity, and shift stressors that are difficult to balance and account for. One study, in particular, involved only a male paramedic cohort [22]. The variation in shift patterns, comparator populations, results, and questionable validity of patient-reported outcomes from a wide variety of assessment tools created a large number of responses that were difficult to interpret and combine.

Following the search strategy developed for this review, we used four specific databases. Other databases or sources may contain literature relevant to the research area in question. Restricting the search to publications in English was due to time constraints for the review, though relevant literature may have been missed due to this restriction. There are other important factors outside of shift schedules that significantly influence fatigue management in EMS. For example, intra-shift variables like napping do affect aspects of shift scheduling variations, and a review designed around provisions for intra-shift sleep recovery potentially could be an important tool to mitigate negative health outcomes related to shift work in EMS [34].

## Conclusions

Previous research has established the relationship between shift work in EMS and increasing fatigue, stress, depression, and adverse health outcomes in EMS personnel. Clinical implications of existing research include the potential for increased fatigue, increased risk of adverse health events, and decreased productivity and task performance in EMS personnel. Gaps in knowledge related to EMS personnel health outcomes and potential associations with workplace scheduling policy exist. Though specific studies show links between shift schedule patterns and such changes in EMS personnel, there is a lack of high-quality evidence available to suggest optimal shift schedule patterns specific to the psychological and physiological demands in EMS. Further study is necessary to provide direct comparisons between shift schedule patterns that reflect the operational requirements in modern EMS systems and characterize both the short and long-term health effects of specific shift schedule patterns.
